# SleepPathfinder: A Socratic Questioning and Self-Decision–Based Chatbot to Support User Engagement in Digital CBT-I: Usability and Feasibility Study

**DOI:** 10.2196/79242

**Published:** 2026-06-09

**Authors:** Youjin Roh, Anderson Sungmin Yoon, Hayoung Oh

**Affiliations:** 1 Department of Applied Artificial Intelligence, Sungkyunkwan University, #32541, Dasan Hall of Economics, Humanities and Social Sciences Campus, Seoul, Republic of Korea, 82 2-740-1785, 82 2-740-1785; 2 Department of Social Welfare, Sungkyunkwan University, Seoul, Republic of Korea

**Keywords:** cognitive behavioral therapy, digital health, CBT, conversational agent, user engagement

## Abstract

**Background:**

Chronic insomnia is a highly prevalent sleep disorder that adversely affects quality of life and mental health. Cognitive behavioral therapy for insomnia (CBT-I) is internationally recommended as the first-line treatment, and digital CBT-I (dCBT-I) has been developed to improve accessibility and scalability. While existing dCBT-I systems effectively support structured behavioral training through standardized protocols, they provide relatively limited support for users’ cognitive exploration and meaning-making processes, particularly in helping users reflect on and internalize the rationale behind CBT-I practices in daily life. These limitations may contribute to challenges in sustained engagement and long-term adherence.

**Objective:**

This study aimed to examine the usability and feasibility of SleepPathfinder, a conversational CBT-I support chatbot that integrates Socratic questioning and a self-decision mechanism to support users’ understanding of and engagement with CBT-I practices.

**Methods:**

SleepPathfinder was designed around a 4-stage conversational flow: education on CBT-I techniques, Socratic cognitive exploration, self-decision, and advice provision. We conducted (1) a single-session pilot usability study (n=45) to assess system stability and user experience and (2) a 5-day condition-based comparative experiment (n=30) consisting of daily sessions, comparing an exploratory dialogue condition with a directive, protocol-guided dialogue condition. Quantitative measures assessed usability, cognitive appraisals related to sleep problems, autonomy-related experiences, and behavioral readiness, while qualitative feedback and conversational log analyses were used to examine interaction patterns and engagement characteristics.

**Results:**

In the comparative experiment, the exploratory dialogue condition showed a tendency toward reduced perceived threat and severity appraisal of sleep problems compared with the directive condition, accompanied by moderate effect sizes in cognitive perception measures. Autonomy-related experiences, including perceived choice and engagement, demonstrated suggestive upward trends in the exploratory condition. Behavioral intention changes were comparable across conditions, while overall readiness for change increased across participants. Conversational log analyses indicated that greater depth and volume of user self-narrative were associated with larger shifts in cognitive appraisals, whereas the frequency of chatbot questions alone was not. The pilot usability study indicated generally positive evaluations of system usability and content credibility, while identifying areas for improvement in emotional responsiveness and conversational naturalness.

**Conclusions:**

These findings suggest that a Socratic questioning–based and self-decision–based conversational structure is usable and feasible as a supportive interaction layer within dCBT-I systems. Rather than altering the directive behavioral structure of CBT-I, such an approach may complement existing protocols by facilitating cognitive exploration and supporting user-perceived autonomy. This study provides design-oriented evidence to inform the refinement of dialogue-supported digital CBT-I systems aimed at enhancing user engagement with CBT-I practices.

## Introduction

Sleep is an essential restorative process that supports physical and mental health, including cognitive functioning, emotional regulation, immune response, metabolic regulation, and overall quality of life [1-4]. However, insomnia is increasingly prevalent in modern society, with approximately 30%‐50% of adults reporting insomnia symptoms and 10%‐15% experiencing clinically significant impairment and distress [5-7]. Chronic insomnia is associated with elevated risks of depression and anxiety [2-4], cardiometabolic diseases [1], and reduced subjective well-being [8-10], and it may function both as a consequence and a predictor of broader mental health problems [3]. These characteristics highlight the importance of early and sustained intervention.

Prior research has identified repetitive and maladaptive cognitive patterns, such as rumination and worry, as key mechanisms in the maintenance of chronic insomnia [11-16]. These cognitive processes exacerbate sustained arousal and anxiety, interfere with sleep initiation, and reinforce negative beliefs about sleep, thereby perpetuating a vicious cycle of insomnia. Addressing these cognitive patterns requires not only the application of behavioral treatment components but also support for individuals to recognize, interpret, and actively engage with their own sleep-related thoughts and behaviors.

Cognitive behavioral therapy for insomnia (CBT-I) is internationally recommended as the first-line treatment for chronic insomnia [17-21], with pharmacological treatment advised only as a short-term adjunct when CBT-I alone is insufficient [22]. CBT-I targets maladaptive beliefs about sleep and conditioned arousal, and prior studies have shown that cognitive changes achieved through CBT-I are relatively stable after treatment completion [17]. In addition to improving sleep-related outcomes, CBT-I has been associated with reductions in depressive and anxiety symptoms and improvements in quality of life [19,20]. CBT-I is typically structured as a multicomponent treatment protocol that includes sleep restriction, stimulus control, sleep hygiene, and cognitive therapy [23]. Despite its strong evidence base, the real-world utilization of CBT-I remains limited due to high costs, shortages of trained therapists, and low referral rates in clinical practice [24-27].

To address these accessibility barriers, digital CBT-I (dCBT-I) has been developed to deliver CBT-I through web- and mobile-based platforms while preserving its core therapeutic components [28-31]. Meta-analytic evidence suggests that dCBT-I can achieve outcomes comparable to face-to-face CBT-I across key sleep-related measures, including insomnia severity, sleep efficiency, and sleep onset latency [29]. Representative systems such as SHUTi, Sleepio, Somzz, and WELT-I implement standardized CBT-I protocols using automated sessions, sleep diaries, and structured behavioral assignments [32-35]. However, studies consistently report low treatment adherence in real-world settings, with many users failing to complete the full treatment program [32-35].

Previous work suggests that this adherence challenge is not primarily due to the standardized or protocol-driven nature of CBT-I itself, but rather to the difficulty users experience in cognitively understanding, accepting, and sustaining engagement with CBT-I practices in daily life. While CBT-I relies on fixed behavioral principles that are well suited for structured training and repeated practice, effective engagement also depends on how users interpret the rationale behind these practices, respond to discomfort or resistance, and integrate them into their lived experiences. In digital environments, opportunities to support users in exploring their individual cognitive interpretations, emotional responses, and readiness for behavior change through personalized dialogue remain relatively limited. As a result, many dCBT-I systems offer only constrained interactive support for facilitating cognitive meaning-making and maintaining motivation alongside ongoing behavioral practice.

Recent advances in natural language–based conversational systems provide opportunities to extend interactive support in digital health interventions through dialogue [36]. Prior studies have shown that conversational interaction can enhance user engagement, emotional acceptance, and perceived support compared with non-dialogue-based digital interventions [37-39]. However, despite these advances, most existing dCBT-I systems have not systematically integrated dialogue-based mechanisms to support cognitive exploration, user-driven reflection, and engagement maintenance within established CBT-I treatment structures.

In this study, we propose *SleepPathfinder*, a conversational CBT-I support chatbot designed to assist users in understanding and engaging with CBT-I treatment guidelines within digital environments. Rather than replacing standardized CBT-I protocols, SleepPathfinder functions as an assistive system that integrates Socratic questioning and a self-decision mechanism to support users in independently exploring their sleep-related cognitions, connecting these insights to directive CBT-I practices, and sustaining engagement with treatment activities. The system is designed to preserve the structured, protocol-driven nature of CBT-I while augmenting it with conversational support aimed at promoting cognitive insight, acceptance, and user-perceived autonomy.

SleepPathfinder is intended for individuals experiencing insomnia-related difficulties in daily life, including those who report persistent sleep problems without necessarily seeking or receiving a formal clinical diagnosis. By clarifying this scope, the system emphasizes early cognitive engagement with structured CBT-I practices rather than delivering stand-alone therapeutic treatment.

The objective of this study is to examine the initial usability, engagement characteristics, and engagement-support potential of a Socratic questioning–based and self-decision–based CBT-I support chatbot. Through a pilot usability study and a short-term, condition-based user experiment, we investigate how conversational interaction may support users’ experiences of engaging with CBT-I practices in digital contexts.

## Methods

### System Overview and Design Rationale

The chatbot system was designed to address challenges related to sustained user engagement and adherence that have been repeatedly reported in real-world use of dCBT-I programs. Prior dCBT-I systems effectively deliver core CBT-I components—such as sleep restriction, stimulus control, sleep hygiene, relaxation techniques, and cognitive restructuring—through standardized, protocol-driven structures that support structured behavioral training and repeated practice [24,25,27,29,30]. While these fixed designs are well suited for implementing evidence-based CBT-I procedures, they offer relatively limited support for helping users cognitively understand, accept, and integrate the rationale behind CBT-I practices into their daily experiences. Difficulties in meaning-making, motivational alignment, and sustained engagement during treatment practice have been associated with reduced adherence and inconsistent long-term use in real-world dCBT-I settings [25,26].

To address these challenges, the system was designed as a conversational support tool that complements standardized CBT-I protocols rather than replacing them. The design rationale was to provide interactive support during the phase in which users form understanding, motivation, and readiness to engage with CBT-I practices. Socratic questioning is used to facilitate the structured exploration of users’ automatic thoughts and emotional responses, while a self-decision mechanism positions users as the primary agents who determine whether and when to engage with specific CBT-I techniques. The dialogue system reflects users’ utterances and contextual cues to support continuity across conversation turns.

**Figure 1.**
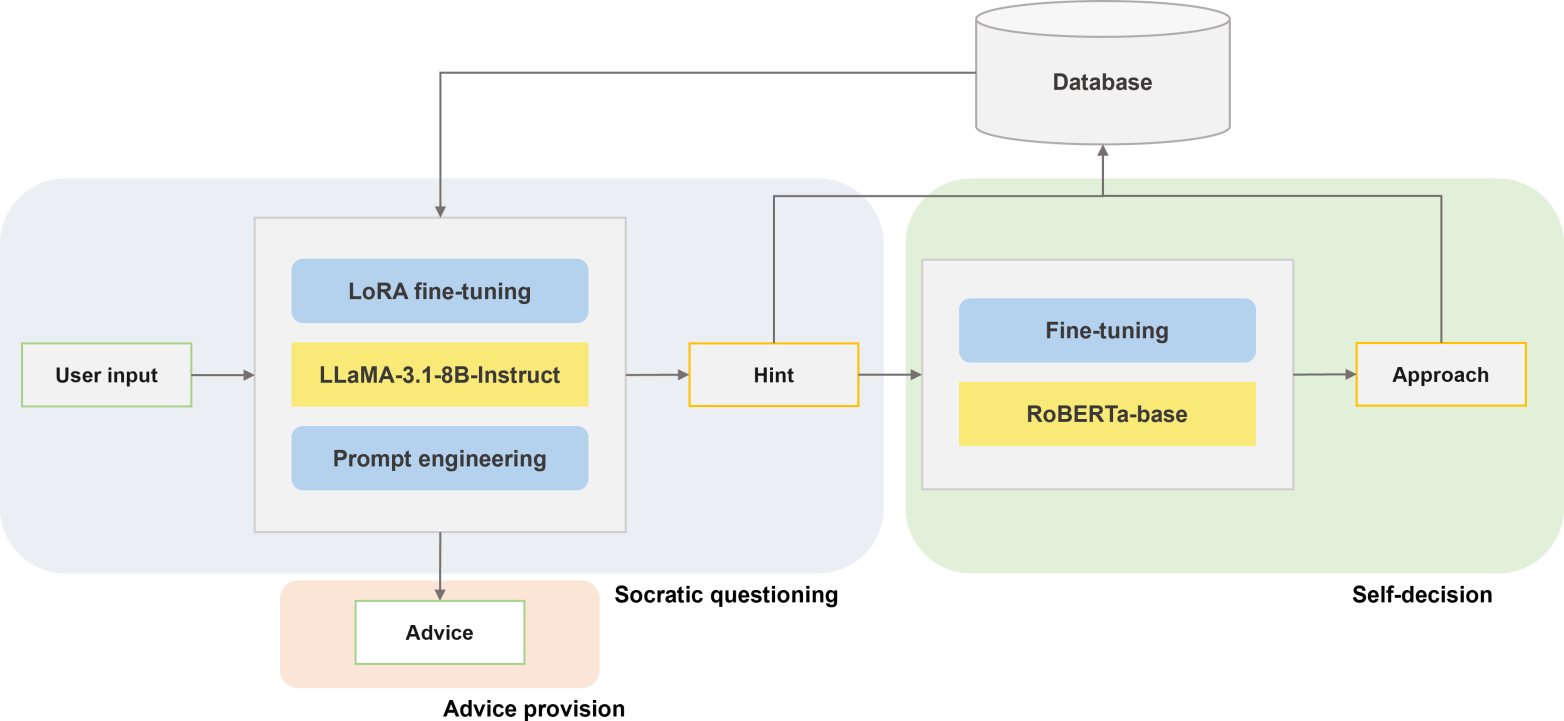
Overall architecture of the CBT-I support chatbot, illustrating how an LLM-based Socratic questioning module and a classifier-based self-decision module interact to support conversational flow and guideline-aligned advice provision. CBT-I: cognitive behavioral therapy for insomnia; LLM: large language model; LoRA: low-rank adaptation; RoBERTa: Robustly Optimized BERT Approach.

The overall system architecture (Figure 1) consists of five core modules that collectively support conversational exploration, user-driven decision-making, and guideline-aligned advice provision within a CBT-I framework: (1) a dialogue generator that produces Socratic questions and empathic responses using a large language model (LLM), (2) an intent classifier that maps user utterances to CBT-I techniques to support the self-decision process, (3) a retrieval module that identifies clinically grounded counseling cases to support advice generation, (4) education templates containing clinically reviewed CBT-I technique content, and (5) an interface layer that enables interaction through button-based selection and multiturn dialogue.

**Figure 2.**
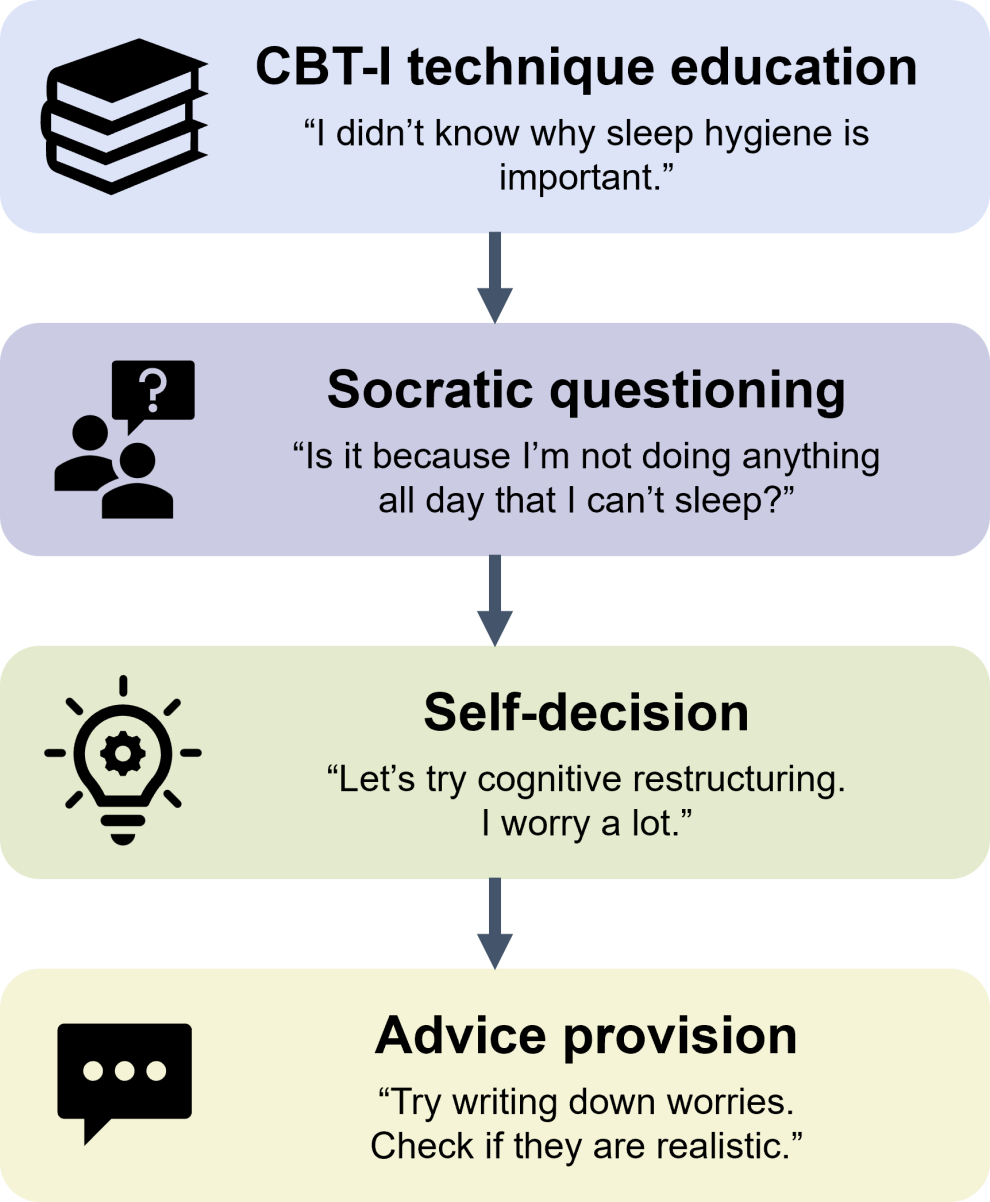
Conversational structure of the CBT-I support chatbot, illustrating transitions from CBT-I technique education to advice provision through Socratic questioning and self-decision. CBT-I: cognitive behavioral therapy for insomnia.

The conversational interaction is structured into 4 sequential stages—education on CBT-I techniques, Socratic questioning, self-decision, and advice provision—as illustrated in Figure 2. This staged design clarifies how standardized CBT-I content is progressively connected to personalized engagement support through dialogue.

### Chatbot Design and Conversational Components

The chatbot operates through a 4-stage conversational flow (education on CBT-I techniques → Socratic questioning → self-decision → advice provision). Each stage has a distinct functional objective and interaction logic, while maintaining continuity such that cognitive and emotional contexts established in earlier stages inform subsequent dialogue. Although a single dialogue engine is used across stages, input handling, prompt structure, output objectives, and interface design are differentiated according to the role of each stage.

#### Education on CBT-I Techniques

The education stage establishes a foundational understanding of core CBT-I techniques and prepares users for subsequent cognitive exploration and decision-making. Five CBT-I techniques—sleep restriction, stimulus control, sleep hygiene, relaxation techniques, and cognitive restructuring—are presented as selectable options within the interface. Upon selection, clinically reviewed educational content based on established guidelines, including objectives, implementation principles, and illustrative examples, is displayed through static templates [40,41].

Generative dialogue is intentionally withheld at this stage to allow users to explore information at their own pace without conversational overload. This design supports cognitive readiness by enabling users to form an initial conceptual link between their sleep difficulties and relevant CBT-I techniques. The content structure and representative message templates for each technique are provided in Table 1.

**Table 1.** Descriptions of the 5 CBT-I^a^ techniques presented by the support chatbot during the CBT-I technique education stage.

CBT-I^a^ element	Description
Sleep restriction	Intentionally limits time in bed to match actual sleep duration, rebuilding the association between bed and sleep. Time in bed is gradually extended as sleep efficiency improves.
Stimulus control	Trains the brain to associate the bed only with sleep by leaving the bed if unable to sleep and returning only when sleepy.
Sleep hygiene	Encourages healthy habits such as maintaining a consistent sleep schedule, avoiding caffeine late in the day, minimizing screen exposure, and reducing stimulating evening activities.
Relaxation techniques	Reduces physical and mental tension using breathing exercises, meditation, light stretching, or progressive muscle relaxation.
Cognitive restructuring	Identifies and reframes negative or distorted thoughts about sleep (eg, “If I don’t sleep well tonight, tomorrow will be ruined”).

a CBT-I: cognitive behavioral therapy for insomnia.

#### Socratic Questioning

The Socratic questioning stage supports structured exploration of users’ automatic thoughts and emotional responses related to sleep. Questions were designed based on 5 categories proposed by Paul and Elder [42]: clarification, assumption probing, evidence exploration, consequence exploration, and alternative exploration. Representative examples of each category are presented in Table 2.

**Table 2.** Socratic question types used in the CBT-I^a^ support chatbot, including their therapeutic purpose and representative example prompts adapted from the framework proposed by Paul and Elder [42].

Question type	Description	Example prompt
Clarification	Probes ambiguities or unclear aspects of a statement	What do you mean by ...?
Probing assumptions	Examines the underlying assumptions behind a belief or statement	Why do you assume ...?
Probing reasons	Seeks justification or supporting evidence for a claim	How did you know ...?
Probing implications	Explores possible consequences or outcomes of a belief	If ..., what might happen as a result?
Exploring alternatives	Considers other perspectives or possible explanations	What else should we consider ...?

a CBT-I: cognitive behavioral therapy for insomnia.

For question generation, the LLaMA 3.1–8B-Instruct model [43] was fine-tuned using the SocratiQ dataset [44] with a low-rank adaptation approach [45]. The original dataset was reformatted into an instruction-input-output schema to support structured Socratic question generation. An example of this instruction-input-output structure is provided in Table 3 to illustrate how abstract Socratic question categories were operationalized into concrete, model-trainable prompts and responses.

**Table 3.** Example of an instruction-input-output dataset sample used for training the Socratic questioning module of the CBT-I^a^ support chatbot.

Instruction	Generate a Socratic question of type “clarity”
Input	However, I would point to the fact that depression/bipolar disorder uses a combination of willpower, therapy, and medication to be cured. There is no cure for bipolar disorder. It is chronic, lifelong, and can only be treated and managed.
Output	Are sleep issues a mental disorder?

a CBT-I: cognitive behavioral therapy for insomnia.

The model generates structured outputs consisting of empathic acknowledgment, question type, subquestion, and a depth-control signal, supporting conversational continuity while encouraging hypothesis testing rather than directive correction [46]. For example, when a user expresses a catastrophic belief about sleep outcomes, the system generates probing questions to facilitate cognitive reappraisal. Full prompt templates are provided in Multimedia Appendix 1.

#### Self-Decision

The self-decision stage is designed to support users’ engagement with standardized CBT-I guidelines by facilitating understanding and acceptance prior to behavioral practice. Rather than altering or personalizing treatment protocols, this stage allows users to consider whether they are ready to engage with a recommended CBT-I technique as a focus for practice. A RoBERTa (Robustly Optimized BERT [Bidirectional Encoder Representations From Transformers] Approach)-based intent classifier [47] maps the user’s most recent utterance to one of the 5 CBT-I techniques and presents this mapping as a recommendation to support reflection, not as a prescription or treatment decision.

This design is informed by self-determination theory (SDT), which highlights the role of perceived autonomy in sustaining engagement and adherence to prescribed behaviors [48-50]. In the context of CBT-I, which is inherently directive and standardized, SDT is not used to modify treatment content but to explain why users may differ in how they understand, accept, and adhere to the same behavioral instructions. Prior studies suggest that when individuals are supported in acknowledging and endorsing prescribed actions, their sense of responsibility for engaging in those actions may increase [51,52].

The intent classifier was trained using 8400 anonymized utterances collected from online sleep-related communities (eg, r/insomnia, r/sleepdisorders), with personally identifiable information removed prior to labeling. To convert community-style posts into consultation-appropriate user statements, we used GPT-4o mini (OpenAI) in an offline preprocessing step to rewrite each post into a first-person, one-on-one conversational utterance while preserving the original concern and emotional tone and removing or generalizing personally identifiable information. The rewriting prompt and annotation prompt specifications are provided in Multimedia Appendix 1.

Each utterance was annotated with an appropriate CBT-I technique using the GPT-4o mini during an offline preprocessing step, and class imbalance was addressed through meaning-preserving data augmentation to yield approximately 2000 samples per technique. During prompt development, multiple candidate prompts were iteratively tested on a subset of posts, and generated outputs were manually reviewed by the research team to ensure preservation of the original concern and appropriate conversational framing. The final prompt was selected based on qualitative inspection of these outputs.

Model training was conducted using k-fold cross-validation, with the best-performing model from each fold selected based on validation performance and saved for subsequent use. The classifier was trained for 3 epochs using AdamW (learning rate = 2 × 10⁻⁵, batch size=8, weight decay=0.01). The RoBERTa-based intent classifier achieved an overall classification accuracy of 0.81 under cross-validation, indicating adequate technical reliability for supporting the self-decision process rather than for autonomous clinical decision-making.

Users may accept the system’s recommendation or defer engagement without restriction. The system does not monitor or enforce behavioral compliance; instead, it functions as a supportive facilitator that helps users cognitively commit to guideline-based actions at their own pace. By positioning this stage after Socratic questioning, the system ensures that behavioral practice follows a process of understanding and acceptance rather than immediate instruction delivery.

Users may accept the system’s recommendation or freely select an alternative CBT-I technique without restriction. This design reflects the self-decision principle, which emphasizes perceived choice and psychological ownership as important factors shaping users’ motivation to engage with prescribed practices [53]. Rather than enforcing compliance, the system is intended to support informed decision-making during users’ early engagement with CBT-I guidelines.

Through this structure, the self-decision stage serves as an interaction-level bridge between cognitive insight and action-oriented readiness within cognitive behavioral therapy (CBT)–based frameworks. Specifically, it supports users in translating the understanding developed through Socratic questioning into a concrete judgment about whether they are ready to engage with a given CBT-I practice, without assuming or enforcing actual behavioral execution [54].

#### Advice Provision

In the final stage, the chatbot provides actionable guidance aligned with the CBT-I technique selected during the self-decision stage. To reduce the risk of hallucinated or context-incongruent responses, a retrieval-augmented generation (RAG) approach was adopted [55]. A Facebook AI Similarity Search (FAISS)–based embedding retrieval system [56] identifies counseling cases with high semantic similarity to the user’s current concern. The RAG dataset consists of 217 expert-curated counseling pairs (client concern–CBT-I technique–counselor response) collected from publicly available online counseling resources. Each case was manually reviewed to ensure alignment with standardized CBT-I techniques. All identifiable information was removed, and each item was labeled according to the CBT-I technique. Dataset structure and representative examples are presented in Table 4.

**Table 4.** Example entries from the 1:1 paired advice generation dataset, consisting of client concerns, corresponding CBT-I^a^ elements, and expert-authored counseling responses used as grounding cases in the retrieval-augmented generation process.

Client concern	CBT-I element	Therapist response
I can’t fall asleep easily at night due to insomnia. I often can’t sleep until dawn. So I end up sleeping during the day and staying awake at night. My friends are concerned about my irregular sleep habits. Is taking naps to make up for lost sleep a good idea? Is there any way to recover from insomnia without relying on medication?	Sleep hygiene	One reason your insomnia might not improve is that your sleep hygiene practices may not align with best practices. Sleeping during the day makes it harder to sleep at night. Even if you didn’t sleep well the night before, try to avoid taking long naps during the day and aim to go to bed and wake up at the same time daily. Also, refrain from using electronic devices before bedtime and avoid eating or exercising too close to bedtime. Keeping a regular routine, especially with exposure to natural light during the day and maintaining a dark environment at night, can help regulate your sleep-wake cycle.
I want to resolve my insomnia...It gets worse as the weekend approaches. I tend to stay up very late and wake up late. I’m also worried that my irregular sleep pattern might be affecting my hormonal cycle.	Sleep restriction	When you frequently change your sleep schedule, especially on weekends, your biological clock gets disrupted, which can worsen insomnia. Sleep restriction aims to stabilize your sleep pattern by limiting the time you spend in bed to the actual time you sleep. This helps increase sleep efficiency. Try to maintain a consistent sleep-wake time, even on weekends. It might be helpful to temporarily reduce time spent in bed and gradually increase it as your sleep becomes more consolidated. Would you be able to try setting a fixed sleep-wake time starting tonight?

a CBT-I: cognitive behavioral therapy for insomnia.

During response generation, the dataset is first filtered by the selected CBT-I technique. The user’s most recent concern is embedded and matched against the filtered dataset using a FAISS-based L2 distance index, and the top three most similar cases (top-k=3) are retrieved based on embedding similarity. No explicit similarity threshold was applied; instead, a fixed top-k strategy was adopted to ensure stable contextual grounding even when user utterances were brief, underspecified, or heterogeneous in wording. Corresponding counselor responses are inserted into the prompt as contextual grounding, guiding the LLM to generate advice consistent with guideline-aligned counseling examples. When “cognitive restructuring” is selected, retrieved cases emphasize question-based exploration rather than behavioral instruction, supporting continued Socratic dialogue [57,58].

### Pilot Study

#### Study Design and Procedure

The pilot study was conducted to examine the technical stability, procedural feasibility, and initial usability of the proposed Socratic questioning–based and self-decision–based CBT-I chatbot, and to identify system-level refinements prior to the main experiment. The study used a single-arm, single-session usability design, consistent with feasibility-focused evaluations of early-stage digital health interventions.

Participants completed one individual chatbot session via a web-based interface, with an average session duration of approximately 15‐20 minutes. The session followed the same 4-stage conversational flow used in the main system: (1) education on CBT-I techniques, (2) Socratic questioning, (3) self-decision, and (4) advice provision. In the education stage, participants selected one of five CBT-I techniques and reviewed the corresponding information. During the Socratic questioning stage, participants engaged in multiple rounds of question-answer interaction to structure their sleep-related concerns. In the self-decision stage, a RoBERTa-based classifier generated a recommended CBT-I strategy, which participants could accept or defer. Finally, the advice provision stage delivered technique-specific behavioral or cognitive guidance.

Immediately after completing the session, participants completed a postsession survey lasting approximately 10 minutes. All sessions were conducted using participants’ personal devices in quiet environments. Data collected in this pilot study were used exclusively to inform system refinement and optimization of the experimental design.

#### Participants

A total of 45 undergraduate and graduate students participated voluntarily (mean age 27.4, SD 8.5 years; 31/45 [68.9%] are female). Academic backgrounds included social welfare and psychology (22/45, 48.9%), engineering and artificial intelligence (AI)–related fields (12/45, 26.7%), and other humanities and social sciences disciplines (11/45, 24.4%). All individuals received an information sheet describing the study purpose, procedures, and data protection measures and provided written informed consent prior to participation. The study was approved by the Institutional Review Board of Sungkyunkwan University (SKKU 2025-03-013), and all data were anonymized. Participants were informed that they could withdraw at any time without penalty and received monetary compensation for their participation.

#### Measures

Pilot evaluation focused on usability, perceived cognitive load, engagement, and overall satisfaction. Quantitative assessment used a 35-item questionnaire with 5-point Likert-scale responses, partially adapted from the System Usability Scale (SUS) [59], NASA Task Load Index (NASA-TLX) [60], User Engagement Scale (UES) [61], and Customer Satisfaction Score (CSAT) [62]. One NASA-TLX item with mixed polarity was excluded, resulting in 34 items. Negatively worded items were reverse-coded prior to analysis.

Qualitative feedback was collected through 3 open-ended questions assessing overall impressions, perceived strengths, and areas for improvement. Responses were analyzed using an inductive content analysis approach. Initial pattern identification was supported by GPT-4o, which was used to assist in grouping responses based on surface-level semantic similarity. The exact prompt used for this assistance is provided in Multimedia Appendix 1. Importantly, GPT output was used solely as an organizational aid and did not replace the researcher’s judgment.

Following AI-assisted clustering, the first author conducted a systematic manual review of all responses to verify grouping consistency and refine cluster labels while preserving participants’ original wording. The qualitative analysis aimed to identify recurring usability and interaction issues relevant to system refinement rather than to generate theory.

#### Analysis

Quantitative analyses consisted of descriptive statistics (means, SDs, and positive response rates) for all questionnaire items. Qualitative responses were analyzed using an inductive thematic approach, in which recurring meaning units were identified and grouped into higher-level categories based on semantic similarity. All qualitative responses were reviewed and coded by a single researcher using an inductive approach, consistent with the exploratory and formative purpose of the pilot usability study. An LLM (GPT-4o mini) was used in a limited capacity to assist with preliminary organization of response excerpts; final theme identification and interpretation were conducted by the researcher. The pilot study was not designed to test hypotheses or estimate intervention effects; rather, findings were used to guide iterative improvements to dialogue flow, system responsiveness, and interface usability prior to the experimental study. All quantitative and qualitative analyses were conducted using Python (version 3.9).

### Experimental Study

#### Research Design

The experimental study was conducted to explore preliminary patterns of cognitive, behavioral, and experiential change associated with the proposed chatbot following refinements informed by the pilot study. In contrast to the pilot implementation using LLaMA 3.1–8B, the experimental system used the GPT-4o mini model to enhance response stability during multiday usage. Core prompt structures and the RoBERTa-based intent classification logic were retained, while dialogue continuity and emotional stability were improved.

Participants were assigned to either an exploratory dialogue condition or a directive dialogue control condition. The exploratory condition experienced the full Socratic questioning → self-decision → advice conversational flow, whereas the control condition received a simplified, directive, protocol-guided CBT-I dialogue without structured Socratic exploration. Participants completed 1 session per day over a 5-day period, with session timing self-selected by participants to reflect naturalistic use conditions in daily life.

The study objectives were exploratory in nature (1) to examine changes in cognitive appraisal of sleep problems, (2) to assess shifts in behavioral intention toward CBT-I practices, and (3) to compare user experience characteristics between exploratory and directive dialogue styles.

#### Participants and Procedure

Thirty participants (14 males, 16 females; mean age 26.4, SD 3.7 years) were recruited and evenly assigned to the two conditions (n=15 per group). Baseline insomnia severity was assessed using the Insomnia Severity Index (ISI-7), with a mean baseline score of 15.63, corresponding to moderate insomnia symptoms. Participants were recruited from a general university student population and were not selected based on predefined insomnia-related inclusion criteria. Insomnia severity was assessed descriptively after recruitment to characterize baseline sleep-related symptoms within the sample, and no formal clinical diagnosis of insomnia was required for participation. Baseline ISI scores were used solely to describe sample characteristics and were not treated as outcome variables in the experimental analyses.

The study procedure consisted of three phases: (1) a pre-intervention survey, (2) five consecutive days of chatbot interaction, and (3) a postintervention survey. Pre- and postsurveys assessed sleep status, cognitive perceptions, behavioral intentions, emotional characteristics, and user experience. Conversation logs were recorded throughout the intervention period, including dialogue length, turn counts, question frequency, and acceptance of recommended strategies.

#### Measures

Cognitive perceptions were assessed using the health belief model (HBM) subscales (perceived severity, perceived susceptibility, and self-understanding) [63]. Behavioral intention and readiness toward CBT-I practices were measured using the theory of planned behavior (TPB) constructs, including outcome beliefs, subjective norms, self-efficacy, and intention [64]. The stage of change was assessed on a 5-point ordinal scale.

User experience was examined with reference to SDT, which highlights the importance of perceived autonomy in shaping motivation to engage with recommended practices [65]. Based on this perspective, user experience was evaluated using autonomy-support items derived from the Health Care Climate Questionnaire and the User Experience Questionnaire–Short (UEQ-S) [66]. Emotional characteristics were assessed using the Perth Alexithymia Questionnaire–Short Form (PAQ-S) [67].

#### Statistical Analysis

Analyses focused on exploratory estimation rather than confirmatory hypothesis testing. Mixed-effects models were used to estimate time (pre vs post) × group interactions, with participants specified as random effects. When model convergence was unstable, generalized estimating equations were used as a complementary approach. Baseline ISI scores were included as covariates in all models.

For sets of secondary outcomes, false discovery rate correction was applied using the Benjamini-Hochberg procedure. Effect sizes (Hedges *g* or odds ratios, as appropriate) and 95% CIs were reported alongside *P* values. Given the feasibility-scale sample size (n=30), analyses emphasize effect size estimation and uncertainty rather than statistical significance, and all secondary analyses are interpreted as exploratory.

### Ethical Considerations

The study was approved by the Institutional Review Board of Sungkyunkwan University (SKKU 2025-08-067-001). Written informed consent was obtained from all participants prior to participation, and they were informed of their right to withdraw from the study at any time without penalty. The chatbot did not provide clinical diagnosis or treatment and was explicitly presented as a supportive tool designed to facilitate engagement with digital CBT-I guidelines rather than as a therapeutic intervention. To ensure participant safety, individuals were advised that if they experienced discomfort or psychological distress during the study, they could immediately discontinue participation and contact the research team for support. No automated monitoring or intervention for suicidal ideation was implemented, and the system was clearly described as not intended for emergency use or crisis intervention. All conversation data were anonymized and securely stored in accordance with institutional data protection guidelines. Monetary compensation was provided for participation.

## Results

### Pilot Study Results: Usability and Feasibility

In the pilot study, the chatbot demonstrated generally favorable usability and feasibility. As summarized in Table 5, mean scores indicated moderate-to-high usability (SUS mean 3.67, SD 0.64) and satisfaction (CSAT mean 3.74, SD 0.71). User engagement scores were comparatively lower (UES mean 3.30, SD 0.81), particularly on items related to emotional responsiveness. Cognitive load was moderate (NASA-TLX mean 3.34, SD 0.45).

**Table 5.** Summary of key quantitative metrics from the pilot usability study (N=45).

Scale	Items, n	Mean (SD)
System Usability Scale (SUS)	10	3.67 (0.64)
NASA Task Load Index (NASA-TLX)	5	3.34 (0.45)
User Engagement Scale (UES)	14	3.30 (0.81)
Customer Satisfaction Score (CSAT)	5	3.74 (0.71)

Exploratory analyses were conducted to examine potential associations between demographic variables and usability ratings. A borderline negative association was observed between age and SUS scores (*P*=.05); however, the effect size was small and of limited practical significance. No statistically significant associations were found between usability measures and other demographic variables, including sex and academic background. Given the formative nature of the pilot evaluation, analyses focused primarily on overall scale-level descriptive statistics.

Qualitative feedback revealed several recurring patterns. Participants frequently highlighted the clarity of CBT-I information, structured questioning flow, and practical solution suggestions, describing the system as “easy to understand,” “informative,” and “well structured.” Many appreciated the initial provision of sleep-related information and the attempt to integrate empathy with reflective questioning.

At the same time, users consistently reported concerns related to delayed response times, repetitive phrasing, limited contextual continuity across conversational turns, and language perceived as overly scripted or robotic. Several participants also noted difficulties with mobile readability, screen size, and scrolling behavior. Others indicated that the chatbot occasionally struggled to fully interpret nuanced or metaphorical expressions, leading to abrupt topic shifts or perceived conversational disconnection.

These qualitative findings informed refinements implemented prior to the experimental study, including improvements in response latency, conversational continuity, repetition reduction, and mobile interface readability. Detailed quantitative item distributions are provided in Multimedia Appendices 2-5. The AI-assisted qualitative grouping results, generated using the prompt described in Multimedia Appendix 1, are presented in Multimedia Appendix 6.

### Baseline Characteristics of the Experimental Sample

The mean baseline ISI score was 15.63 (SD 4.06), with approximately half of the participants classified in the moderate insomnia range (50%). No significant demographic imbalances were observed between groups; sex distribution (53% women, 47% men) and age were comparable across conditions. Baseline ISI scores are reported to characterize the sample and were not analyzed as outcome variables.

### Cognitive and Behavioral Outcomes

Among the HBM subscales, perceived severity of sleep problems significantly decreased in the exploratory group, whereas it showed an increasing trend in the directive group, yielding a significant group × time interaction in the mixed-effects model (*β*=0.467, 95% CI 0.078-0.855; *P*=.02; Welch Δ: *g*=0.808). Perceived susceptibility remained relatively stable in both groups. Self-awareness increased over time in both conditions, with no statistically significant between-group difference.

Across TPB constructs—outcome beliefs, subjective norms, self-efficacy, and behavioral intention—scores increased from pre- to postintervention; however, no statistically significant group × time interaction effects were observed. The overall direction of change across TPB constructs was consistent with canonical TPB pathways, indicating increased readiness toward CBT-I practices across conditions.

**Figure 3.**
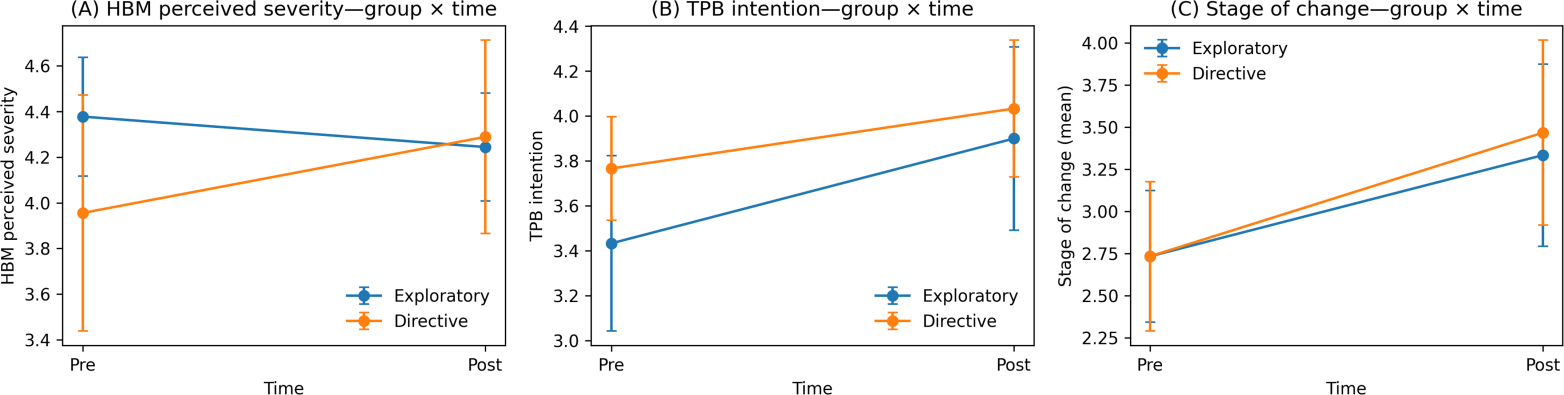
Group × time patterns in cognitive and behavioral measures: (A) changes in perceived severity (health belief model) by dialogue condition, (B) changes in behavioral intention (theory of planned behavior) across groups, and (C) distributional shifts in the stage of change from pre- to postintervention. Error bars indicate 95% CIs. HBM: health belief model; TPB: theory of planned behavior.

Stage of change scores shifted toward higher readiness levels, with the mean stage moving from contemplation (2) toward preparation (3) or higher. The proportion of participants reaching the preparation stage or above was comparable across groups (exploratory: 80%; directive: 93%). Baseline stage significantly predicted postintervention stage (ordered logit: odds ratio 3.16, 95% CI 1.12-8.92; *P*=.03). Figure 3 illustrates changes in key cognitive and behavioral outcomes by group over time.

A comprehensive summary of primary and key secondary outcomes, including effect sizes and multiple-comparison–adjusted results, is presented in Multimedia Appendix 7.

### Autonomy and User Experience

SDT-based autonomy scores were higher in the exploratory group and showed marginal significance after covariate adjustment (ANCOVA *P*=.07, η²=0.168). No statistically significant group differences were observed in overall UEQ-S scores. However, hedonic quality scores were higher in the exploratory group (Hedges *g*=0.435), although this difference did not reach statistical significance after adjustment (ANCOVA *P*=.18).

**Figure 4.**
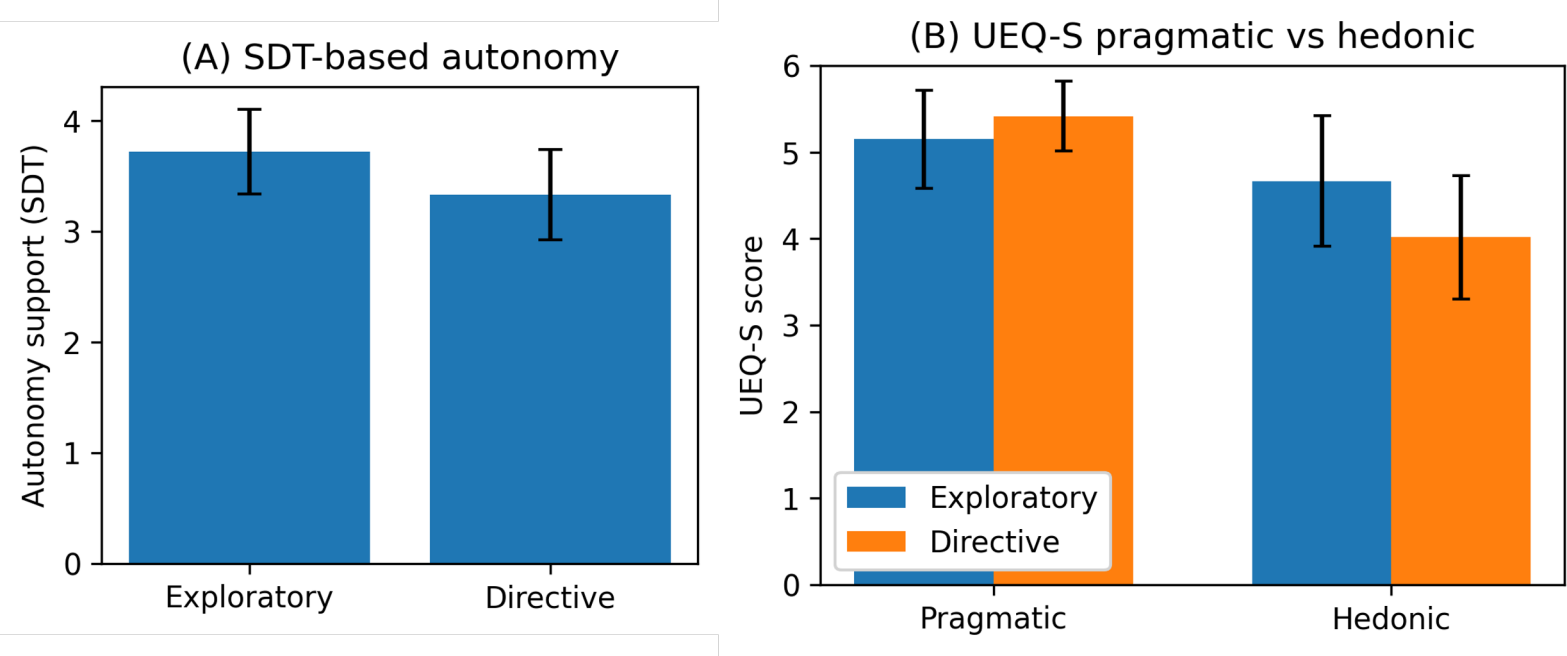
Group differences in user experience measures by dialogue condition: (A) autonomy support scores based on self-determination theory (SDT) and (B) pragmatic and hedonic quality scores from the User Experience Questionnaire–Short (UEQ-S). Error bars indicate 95% CIs.

Figure 4 presents group-level comparisons of autonomy and hedonic quality scores across conditions.

### Moderating Role of Alexithymia

**Figure 5.**
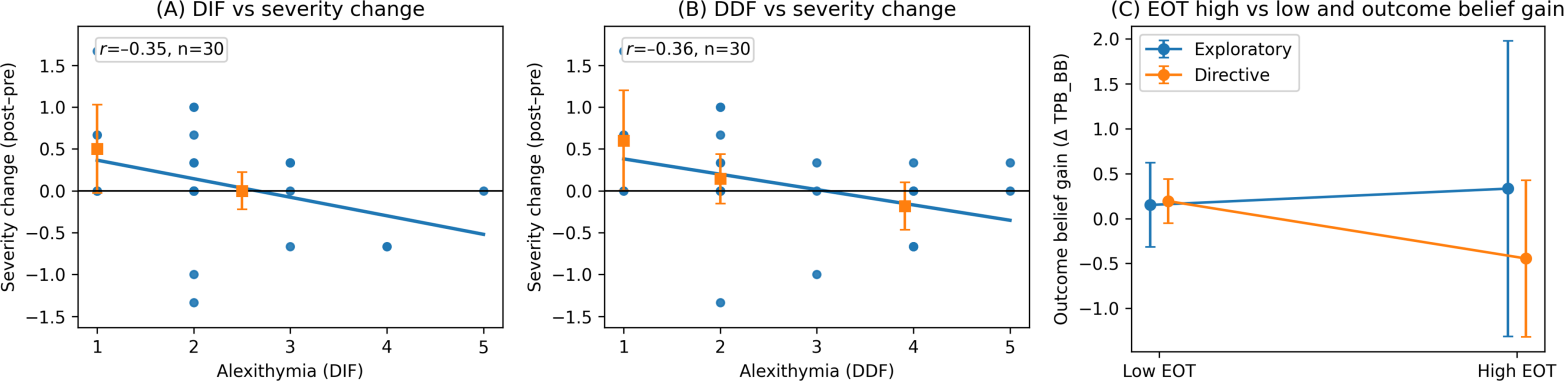
Associations between alexithymia-related traits and changes in cognitive and behavioral measures. (A) Relationship between difficulty identifying feelings (DIF) and change in perceived severity. (B) Relationship between difficulty describing feelings (DDF) and change in perceived severity. (C) Differences in outcome belief change by levels of externally oriented thinking (EOT). Error bars indicate 95% CIs.

Higher levels of difficulty identifying feelings (DIF) and difficulty describing feelings (DDF), as measured by the PAQ-S, were associated with smaller reductions in HBM perceived severity (pooled Pearson *r*=0.35 and 0.36, respectively). In contrast, lower levels of externally oriented thinking (EOT) were associated with greater increases in self-awareness and outcome beliefs. Figure 5 illustrates these associations.

### Log-Outcome Associations

Log-derived engagement metrics showed several associations with cognitive outcomes. Greater use of causal self-explanation (mean_cause_words) was associated with larger reductions in perceived severity of sleep problems (*r*=0.47, *P*=.009). Higher chatbot question frequency (mean_qturns) was associated with smaller magnitudes of change (*r*=0.40, *P*=.03). Fewer turns required to accept advice (turns_to_accept) were associated with greater cognitive change, and longer session duration was associated with larger increases in outcome beliefs.

Comparative log analysis indicated that the exploratory group produced more causal words per session (31.6 vs 15.4) and more words per user turn (3.82 vs 2.83), whereas the directive group exhibited a higher proportion of question-tagged turns (0.208 vs 0.098). Advice acceptance rates were higher in the directive condition (0.292 vs 0.054), while early session termination occurred more frequently in the exploratory condition (0.446 vs 0.000).

## Discussion

### Principal Findings

This study proposed and examined the usability, feasibility, and interaction characteristics of a conversational digital CBT-I support chatbot that integrates Socratic questioning and a self-decision mechanism. The system was not designed to replace standardized CBT-I protocols or to deliver therapeutic content independently; rather, it was intentionally positioned as a dialogue-based assistive layer that supports how users understand, interpret, and engage with established CBT-I practices within digital environments.

Across a pilot usability study and a short-term, 5-day condition-based comparative experiment, the findings suggest that Socratic questioning–based exploratory dialogue can influence users’ cognitive appraisal of their sleep problems. In particular, participants in the exploratory dialogue condition demonstrated a reduction in HBM perceived severity of sleep problems, whereas no comparable pattern was observed in the directive condition. Importantly, this change occurred without a corresponding decrease in perceived susceptibility, indicating a shift in cognitive appraisal rather than disengagement from sleep-related concerns. This pattern aligns with the system’s design goal of supporting cognitive reappraisal while preserving the perceived relevance and seriousness of insomnia-related difficulties.

In addition, the self-decision structure was associated with higher autonomy-related experiences and trends toward enhanced hedonic quality of interaction. In this study, autonomy should not be interpreted solely as a subjective outcome measure, but also as a design construct: the self-decision mechanism explicitly positions users as active agents who may endorse or defer guideline-based actions following cognitive exploration. In this sense, self-decision operationalizes autonomy at the interaction level rather than attempting to personalize or modify CBT-I treatment content itself. Although not all user experience measures reached statistical significance, the observed effect size patterns suggest that enabling users to cognitively commit to prescribed practices may meaningfully shape engagement-related experiences in digital CBT-I contexts.

### Interpretation of Cognitive and Behavioral Patterns

The observed changes in HBM constructs and stage of change scores indicate that the chatbot primarily supported readiness for change rather than immediate behavioral performance. This interpretation is consistent with both the feasibility-oriented nature of the study and the intentionally brief intervention duration. Given that CBT-I typically requires several weeks of repeated behavioral practice—particularly for core components such as sleep restriction and stimulus control—the present findings should not be interpreted as evidence of sleep behavior change or clinical improvement.

Rather, the system appears to have functioned as a cognitive catalyst that reorganized how users conceptualized insomnia and their relationship to CBT-I guidelines. The overall shift toward higher readiness levels suggests that the conversational structure supported preparatory cognitive work—such as reframing perceived threat, clarifying personal relevance, and acknowledging ambivalence—that commonly precedes sustained behavioral adherence in longer-term CBT-I programs.

Within CBT frameworks, Socratic questioning has traditionally been used as a therapeutic technique to facilitate cognitive restructuring by guiding clients to examine automatic thoughts and underlying assumptions. In the present system, this approach was adapted not as a therapeutic intervention in itself, but as a conversational scaffold designed to support early-stage cognitive exploration in a digital context. This distinction is important, as the system was not intended to replicate face-to-face therapeutic interaction, but to provide structured support for users’ sense-making processes during initial engagement with CBT-I practices.

Log-derived analyses further contextualized these findings. Greater depth of user self-narrative and causal self-explanation was associated with larger reductions in perceived severity, whereas higher frequencies of chatbot questioning were associated with smaller magnitudes of change. This pattern suggests that the qualitative coherence and perceived relevance of dialogue may be more influential than the sheer quantity of Socratic prompts, underscoring the importance of carefully calibrated dialogue design rather than aggressive questioning strategies in conversational support systems.

### Design Implications for Digital CBT-I Support

From a design perspective, these findings suggest that digital CBT-I systems may benefit from augmenting existing protocol-driven structures with interaction mechanisms that support cognitive exploration and autonomous judgment. Importantly, this does not imply that fixed, standardized CBT-I modules are inherently problematic. On the contrary, structured behavioral protocols are essential for delivering sleep restriction, stimulus control, and other core CBT-I techniques, and their fixed nature often facilitates usability, consistency, and adherence.

The present findings instead highlight a complementary design space: supporting how users cognitively interpret and accept these standardized practices. Because many CBT-I components require effortful behavioral performance over extended periods, difficulties in adherence may arise not from insufficient instruction, but from users’ uncertainty, resistance, or incomplete understanding of why these practices are necessary. The combination of Socratic questioning and self-decision offers one operational pathway for addressing this gap by supporting users’ cognitive endorsement of prescribed practices prior to execution, while preserving the directive structure of CBT-I itself.

The results also indicate a potential trade-off between structural guidance and cognitive engagement. While the directive condition showed higher advice acceptance rates, exploratory dialogue was associated with greater depth of self-reflection but also higher rates of early disengagement. This polarization suggests that future systems may benefit from adaptive strategies that dynamically balance exploration and guidance based on real-time indicators of user readiness, engagement, and cognitive load.

### Comparison With Prior dCBT-I Systems

Most established digital CBT-I systems, including Sleepio [33], SHUTi [32], CBT-I Coach [31], Somzz [34], and WELT-I [35], deliver treatment through structured, sequential modules emphasizing behavioral prescriptions such as sleep restriction, stimulus control, and sleep diary monitoring. These systems have demonstrated clinical efficacy in randomized controlled trials and meta-analyses and play a critical role in expanding access to evidence-based insomnia treatment.

A recent large-scale meta-analysis of fully automated digital CBT-I including 32 randomized controlled trials reported a moderate-to-large posttreatment effect on insomnia severity (standardized mean difference −0.71, 95% CI −0.88 to −0.54; *P*<.001; *k*=32), although statistical heterogeneity was considerable (*I*²=91%) [68]. Furthermore, therapist-assisted CBT-I has consistently demonstrated stronger effects than fully automated formats, suggesting that supportive or interactive components contribute meaningfully beyond standardized behavioral content delivery.

Systematic reviews examining commercial dCBT-I applications have highlighted the importance of engagement-oriented design. Uyumaz et al [69] observed that most applications incorporate interactive panels, sleep diaries, feedback loops, reminders, goal-setting modules, and motivational components to enhance user engagement and adherence. Despite these design features, adherence remains a persistent challenge in real-world digital behavioral interventions.

Recent clinical evidence suggests that adherence should not be conceptualized solely in terms of module completion. In a randomized controlled trial of smartphone-based dCBT-I, Lee et al [70] demonstrated that behavioral adherence—such as compliance with sleep efficiency targets and sleep restriction recommendations—was more strongly associated with improvements in sleep outcomes than simple completion of modules. Similarly, meta-regression findings from fully automated dCBT-I analyses indicate that completion rate alone does not significantly predict treatment effect size [68], underscoring the importance of qualitative aspects of engagement.

Taken together, these findings identify a specific gap in current digital CBT-I frameworks: while behavioral content is standardized and empirically supported, support for users’ cognitive engagement with these prescriptions—particularly during early exposure and short-term use—remains comparatively limited. Most systems personalize treatment at the level of content sequencing or progress adaptation. In contrast, SleepPathfinder introduces personalization at the level of interaction. Rather than modifying therapeutic guidelines, it supports users in examining their reasoning, emotional resistance, and readiness prior to committing to guideline-based actions.

The observed associations in this study between dialogue depth, self-explanatory language, and changes in cognitive appraisal suggest that interaction-level personalization may address engagement-related challenges identified in prior systematic reviews [69]. By facilitating cognitive meaning-making before behavioral implementation, the system may complement existing dCBT-I frameworks that already provide strong behavioral scaffolding but limited dialogic exploration.

Finally, emerging perspectives on AI- and LLM-supported CBT-I propose that conversational systems may enhance personalization, real-time feedback, and adaptive motivational support [71]. Within this evolving landscape, SleepPathfinder represents an initial step toward integrating structured CBT-I protocols with dialogue-based mechanisms designed to support adherence-related cognitive processes while maintaining the standardized foundation of established digital treatments.

### Limitations and Ethical Considerations

Several limitations should be considered when interpreting these findings. First, the sample size was modest, the intervention period was limited to 5 days, and no clinical sleep outcomes were assessed. Accordingly, the study was not designed to evaluate treatment effectiveness, symptom reduction, or long-term adherence. The pilot study was intentionally designed as a single-session evaluation to assess immediate usability and interaction quality, rather than sustained engagement or behavioral outcomes. Additionally, the system has not undergone clinical validation as a therapeutic intervention, and its effects on formally diagnosed insomnia populations remain to be established in rigorously controlled trials.

Second, participants were recruited from a university population and were not selected based on formal insomnia diagnoses, limiting generalizability to clinical populations. Moreover, recruitment was voluntary, potentially introducing selection bias toward individuals who are more technologically familiar or open to AI-mediated interaction.

Qualitative feedback from the pilot study was analyzed using an exploratory thematic summarization approach rather than a formal qualitative methodology. Thematic grouping was conducted by the research team without independent multirater coding or calculation of interrater reliability. Accordingly, these qualitative findings should be interpreted as descriptive insights intended to inform iterative system refinement rather than as rigorously validated thematic conclusions. Because participants were aware that they were taking part in a study evaluating the system, engagement patterns and reported experiences may have been influenced by observation effects (ie, a Hawthorne effect).

From a technical perspective, some system components exhibited limitations. In particular, generative inference mechanisms used to support Socratic questioning may be vulnerable to misclassification or abstraction errors when applied to open-ended user input. While additional technical validation was conducted to ensure functional reliability of the system components, these mechanisms were not used for clinical decision-making or outcome evaluation. Their limitations, nonetheless, highlight the need for caution when deploying language model–based reasoning tools in mental health–related contexts. Additionally, although retrieval-based grounding was used to reduce hallucination risk, algorithmic biases in dataset composition or embedding similarity may influence which counseling examples are retrieved and emphasized, potentially shaping recommendation framing in subtle ways.

In addition, the intent classification dataset was partially constructed using LLM-based rewriting and annotation of Reddit posts. Although prompts were iteratively refined and outputs were manually reviewed during development, this process may have introduced systematic biases related to language style, community-specific expression patterns, or model-driven categorization. Future work should incorporate more diverse data sources and, where feasible, clinician-reviewed annotations to strengthen external validity.

Ethically, the system was not designed to detect or respond to acute psychological risk, and AI-mediated dialogue introduces risks related to misinterpretation, overdependence, and data privacy. While these risks were mitigated through study design, informed consent, and data anonymization, future deployments will require more robust safeguards, monitoring strategies, and appropriate clinical oversight.

### Future Directions

Future research should evaluate the proposed conversational structure in larger and more diverse samples, including clinically diagnosed insomnia populations, with longer intervention periods and behavior-based outcome measures such as sleep diaries and longitudinal usage logs. Critically, future studies should examine whether conversational support during early engagement phases translates into improved adherence and sustained behavioral practice within full-length CBT-I programs.

Although this study was conducted in nonclinical contexts, the cognitive engagement mechanisms identified here may inform future adaptations for individuals with clinically diagnosed insomnia. In particular, the integration of Socratic questioning and self-decision structures may support how users cognitively interpret and commit to standardized CBT-I protocols in both early-stage and clinical populations.

More broadly, this work suggests that digital CBT-I systems may benefit from being designed not as replacements for therapy, but as usability-oriented interaction layers that support users’ cognitive engagement with established, evidence-based treatment protocols. By clarifying how users make sense of and commit to standardized practices, conversational support systems may play a complementary role in improving real-world uptake and persistence of digital CBT-I interventions.

## Supplementary material

10.2196/79242 Multimedia Appendix 1 Prompt specifications used for annotation, data augmentation, Socratic questioning, confidence estimation, empathic response generation, and qualitative response clustering in the SleepPathfinder system.

10.2196/79242 Multimedia Appendix 2 Item-level mean scores for the System Usability Scale (SUS) (N=45).

10.2196/79242 Multimedia Appendix 3 Item-level mean scores for the NASA Task Load Index (NASA-TLX), reflecting perceived cognitive workload during chatbot interaction (N=45).

10.2196/79242 Multimedia Appendix 4 Item-level mean scores for the User Engagement Scale (UES), reflecting focused attention, perceived usability, aesthetic appeal, and reward (N=45).

10.2196/79242 Multimedia Appendix 5 Item-level mean scores for the Customer Satisfaction Score (CSAT), assessing overall satisfaction with the system’s credibility and perceived usefulness (N=45).

10.2196/79242 Multimedia Appendix 6 Artificial intelligence–assisted exploratory thematic grouping.

10.2196/79242 Multimedia Appendix 7 Key focal and exploratory outcomes with effect sizes, 95% CIs, and multiple-comparison adjustment. Mixed-effects models report fixed-effect estimates (*β*) for group × time interactions, whereas post-only outcomes report covariate-adjusted group effects. Benjamini-Hochberg–adjusted *q* values are reported to control the false discovery rate (*q*=0.10).

10.2196/79242 Multimedia Appendix 8 Item-level descriptive statistics for pilot usability evaluation measures.

## Abbreviations

AI: artificial intelligence
BERT: Bidirectional Encoder Representations From Transformers
CBT: cognitive behavioral therapy
CBT-I: cognitive behavioral therapy for insomnia
CSAT: Customer Satisfaction Score
dCBT-I: digital cognitive behavioral therapy for insomnia
DDF: difficulty describing feelings
DIF: difficulty identifying feelings
EOT: externally oriented thinking
FAISS: Facebook AI Similarity Search
HBM: health belief model
ISI: Insomnia Severity Index
LLM: large language model
NASA-TLX: NASA Task Load Index
PAQ-S: Perth Alexithymia Questionnaire–Short Form
RAG: retrieval-augmented generation
RoBERTa: Robustly Optimized BERT Approach
SDT: self-determination theory
SUS: System Usability Scale
TPB: theory of planned behavior
UEQ-S: User Experience Questionnaire–Short
UES: User Engagement Scale

## References

[1] Watson NF, Badr MS, Belenky G (2015). Joint consensus statement of the American Academy of Sleep Medicine and Sleep Research Society on the recommended amount of sleep for a healthy adult: methodology and discussion. J Clin Sleep Med.

[2] Thumann BF, Börnhorst C, Michels N (2019). Cross-sectional and longitudinal associations between psychosocial well-being and sleep in European children and adolescents. J Sleep Res.

[3] Gehrman P, Seelig AD, Jacobson IG (2013). Predeployment sleep duration and insomnia symptoms as risk factors for new-onset mental health disorders following military deployment. Sleep.

[4] Broberg L, Damm P, Frokjaer VG (2022). Evaluation of the effect of supervised group exercise on self-reported sleep quality in pregnant women with or at high risk of depression: a secondary analysis of a randomized controlled trial. Int J Environ Res Public Health.

[5] Schutte-Rodin S, Broch L, Buysse D, Dorsey C, Sateia M (2008). Clinical guideline for the evaluation and management of chronic insomnia in adults. J Clin Sleep Med.

[6] Roth T (2007). Insomnia: definition, prevalence, etiology, and consequences. J Clin Sleep Med.

[7] Riemann D, Baglioni C, Bassetti C (2017). European guideline for the diagnosis and treatment of insomnia. J Sleep Res.

[8] Cunningham JEA, Shapiro CM (2018). Cognitive behavioural therapy for insomnia (CBT-I) to treat depression: a systematic review. J Psychosom Res.

[9] Schlarb AA, Kulessa D, Gulewitsch MD (2012). Sleep characteristics, sleep problems, and associations of self-efficacy among German university students. Nat Sci Sleep.

[10] Williams AB, Dzierzewski JM, Griffin SC, Lind MJ, Dick D, Rybarczyk BD (2020). Insomnia disorder and behaviorally induced insufficient sleep syndrome: prevalence and relationship to depression in college students. Behav Sleep Med.

[11] Ellis J, Hampson SE, Cropley M (2007). The role of dysfunctional beliefs and attitudes in late-life insomnia. J Psychosom Res.

[12] Yu J, Xiang T, Pan J (2020). The relationship between dysfunctional beliefs and attitudes about sleep and sleep structure in patients with insomnia: a controlled study. Psychology.

[13] Carney CE, Harris AL, Moss TG, Edinger JD (2010). Distinguishing rumination from worry in clinical insomnia. Behav Res Ther.

[14] Clancy F, Prestwich A, Caperon L, Tsipa A, O’Connor DB (2020). The association between worry and rumination with sleep in non-clinical populations: a systematic review and meta-analysis. Health Psychol Rev.

[15] Carney CE, Harris AL, Falco A, Edinger JD (2013). The relation between insomnia symptoms, mood, and rumination about insomnia symptoms. J Clin Sleep Med.

[16] Hvenegaard M, Watkins ER, Poulsen S (2015). Rumination-focused cognitive behaviour therapy vs. cognitive behaviour therapy for depression: study protocol for a randomised controlled superiority trial. Trials.

[17] Thakral M, Von Korff M, McCurry SM, Morin CM, Vitiello MV (2020). Changes in dysfunctional beliefs about sleep after cognitive behavioral therapy for insomnia: a systematic literature review and meta-analysis. Sleep Med Rev.

[18] Ellis JG, Cushing T, Germain A (2015). Treating acute insomnia: a randomized controlled trial of a “single-shot” of cognitive behavioral therapy for insomnia. Sleep.

[19] Alimoradi Z, Jafari E, Broström A (2022). Effects of cognitive behavioral therapy for insomnia (CBT-I) on quality of life: a systematic review and meta-analysis. Sleep Med Rev.

[20] Palagini L, Hertenstein E, Riemann D, Nissen C (2022). Sleep, insomnia and mental health. J Sleep Res.

[21] Rossman J (2019). Cognitive-behavioral therapy for insomnia: an effective and underutilized treatment for insomnia. Am J Lifestyle Med.

[22] Qaseem A, Kansagara D, Forciea MA, Cooke M, Denberg TD, Clinical Guidelines Committee of the American College of Physicians (2016). Management of chronic insomnia disorder in adults: a clinical practice guideline from the American College of Physicians. Ann Intern Med.

[23] Walker J, Muench A, Perlis ML, Vargas I (2022). Cognitive behavioral therapy for insomnia (CBT-I): a primer. Klin Spec Psihol.

[24] Vargas I, Egeler M, Walker J, Benitez DD (2023). Examining the barriers and recommendations for integrating more equitable insomnia treatment options in primary care. Front Sleep.

[25] Conroy DA, Ebben MR (2015). Referral practices for cognitive behavioral therapy for insomnia: a survey study. Behav Neurol.

[26] Koffel E, Bramoweth AD, Ulmer CS (2018). Increasing access to and utilization of cognitive behavioral therapy for insomnia (CBT-I): a narrative review. J Gen Intern Med.

[27] Ulmer CS, Bosworth HB, Beckham JC (2017). Veterans Affairs primary care provider perceptions of insomnia treatment. J Clin Sleep Med.

[28] Denis D, Eley TC, Rijsdijk F (2020). Is digital cognitive behavioural therapy for insomnia effective in treating sub-threshold insomnia: a pilot RCT. Sleep Med.

[29] Soh HL, Ho RC, Ho CS, Tam WW (2020). Efficacy of digital cognitive behavioural therapy for insomnia: a meta-analysis of randomised controlled trials. Sleep Med.

[30] Vollert B, Müller L, Jacobi C, Trockel M, Beintner I (2023). Effectiveness of an app-based short intervention to improve sleep: randomized controlled trial. JMIR Ment Health.

[31] Kuhn E, Weiss BJ, Taylor KL (2016). CBT-I Coach: a description and clinician perceptions of a mobile app for cognitive behavioral therapy for insomnia. J Clin Sleep Med.

[32] Thorndike FP, Saylor DK, Bailey ET, Gonder-Frederick L, Morin CM, Ritterband LM (2008). Development and perceived utility and impact of an internet intervention for insomnia. E J Appl Psychol.

[33] Sampson C, Bell E, Cole A (2022). Digital cognitive behavioural therapy for insomnia and primary care costs in England: an interrupted time series analysis. BJGP Open.

[34] Shin J, Kim S, Lee J (2024). Efficacy of mobile app-based cognitive behavioral therapy for insomnia: multicenter, single-blind randomized clinical trial. J Med Internet Res.

[35] Park KM, Lee S, Lee Y, Moon DU, Lee E (2025). Validating the efficacy of a mobile digital therapeutic for insomnia (WELT-I): randomized controlled decentralized clinical trial. J Med Internet Res.

[36] Siddals S, Torous J, Coxon A (2024). “It happened to be the perfect thing”: experiences of generative AI chatbots for mental health. NPJ Ment Health Res.

[37] Yuan A, Garcia Colato E, Pescosolido B, Song H, Samtani S (2025). Improving workplace well-being in modern organizations: a review of large language model-based mental health chatbots. ACM Trans Manage Inf Syst.

[38] Fitzpatrick KK, Darcy A, Vierhile M (2017). Delivering cognitive behavior therapy to young adults with symptoms of depression and anxiety using a fully automated conversational agent (Woebot): a randomized controlled trial. JMIR Ment Health.

[39] Inkster B, Sarda S, Subramanian V (2018). An empathy-driven, conversational artificial intelligence agent (Wysa) for digital mental well-being: real-world data evaluation mixed-methods study. JMIR Mhealth Uhealth.

[40] Anderson KN (2018). Insomnia and cognitive behavioural therapy-how to assess your patient and why it should be a standard part of care. J Thorac Dis.

[41] Taylor DJ, Peterson AL, Goodie JL (2019). Cognitive-behavioral therapy for insomnia in the military: therapist guide. The University of Arizona.

[42] Paul RW, Elder L (2006). Critical thinking: the nature of critical and creative thought. J Dev Educ.

[43] (2024). Introducing Llama 3.1: our most capable models to date. Meta AI.

[44] Ang BH, Gollapalli SD, Ng SK (2023). Proceedings of the 17th Conference of the European Chapter of the Association for Computational Linguistics.

[45] Hu EJ, Shen Y, Wallis P, Allen-Zhu Z, Li Y, Wang S (2021). LoRA: low-rank adaptation of large language models. arXiv.

[46] Qi J, Xu Z, Shen Y (2023). Proceedings of the 2023 Conference on Empirical Methods in Natural Language Processing.

[47] Liu Y, Ott M, Goyal N, Du J, Joshi M, Chen D (2019). RoBERTa: a robustly optimized BERT pretraining approach. arXiv.

[48] Bäuml J, Froböse T, Kraemer S, Rentrop M, Pitschel-Walz G (2006). Psychoeducation: a basic psychotherapeutic intervention for patients with schizophrenia and their families. Schizophr Bull.

[49] Bashiri Z, Aghajani M, Masoudi Alavi N (2016). Effects of psychoeducation on mental health in patients with coronary heart disease. Iran Red Crescent Med J.

[50] Paranthaman V, Satnam K, Lim JL (2010). Effective implementation of a structured psychoeducation programme among caregivers of patients with schizophrenia in the community. Asian J Psychiatr.

[51] Sousa P, Martinho R, Parreira P, Luo G (2023). Editorial: mHealth tools for patient empowerment and chronic disease management. Front Psychol.

[52] Battista S, Giardulli B, Sieiro Santos C (2024). Digital health and self-management in idiopathic inflammatory myopathies: a missed opportunity?. Curr Rheumatol Rep.

[53] Al-Wahedi M, Ismail S, AlKhofani W, Azman A (2024). Automation of electronic cognitive behavioural therapy (automated-eCBT) in adapting psychotherapy in a clinical context: a review. Psychology (Irvine).

[54] Guo L, Li L, Lu Y (2023). Effects of empowerment education on the self-management and self-efficacy of liver transplant patients: a randomized controlled trial. BMC Nurs.

[55] Lewis P, Perez E, Piktus A, Petroni F, Karpukhin V, Goyal N (2020). Proceedings of the 34th International Conference on Neural Information Processing Systems (NeurIPS).

[56] Douze M, Guzhva A, Deng C (2026). The FAISS library. IEEE Trans Big Data.

[57] Clark GI, Egan SJ (2015). The Socratic method in cognitive behavioural therapy: a narrative review. Cogn Ther Res.

[58] Vittorio LN, Murphy ST, Braun JD, Strunk DR (2022). Using Socratic Questioning to promote cognitive change and achieve depressive symptom reduction: Evidence of cognitive change as a mediator. Behav Res Ther.

[59] Brooke J, Jordan PW, Thomas B, McClelland IL, Weerdmeester B (1996). Usability Evaluation in Industry.

[60] Hart SG, Staveland LE, Hancock PA, Meshkati N (1988). Human Mental Workload.

[61] O’Brien HL, Cairns P, Hall M (2018). A practical approach to measuring user engagement with the refined User Engagement Scale (UES) and new UES short form. Int J Hum Comput Stud.

[62] Fornell C, Johnson MD, Anderson EW, Cha J, Bryant BE (1996). The American Customer Satisfaction Index: nature, purpose, and findings. J Mark.

[63] Crowther ME, Ferguson SA, Gupta CC, Reynolds AC (2024). The development and validation of the Health Belief Model for Shift Workers (HBM-SW) scale. Behav Sleep Med.

[64] Romero Reyes D, Moriano León JA, Ybarra Sagarduy JL (2023). Development and validation of the help-seeking intention scale in university students with hazardous and harmful consumption of alcohol. Front Psychol.

[65] Matin H, Nadrian H, Jahangiry L, Sarbakhsh P, Shaghaghi A (2019). Psychometric properties of the Persian Health Care Climate Questionnaire (HCCQ-P): assessment of type 2 diabetes care supportiveness in Iran. Patient Prefer Adherence.

[66] Schrepp M, Hinderks A, Thomaschewski J (2017). Design and evaluation of a short version of the User Experience Questionnaire (UEQ-S). Int J Interact Multimed Artif Intell.

[67] Preece DA, Mehta A, Petrova K (2023). The Perth Alexithymia Questionnaire-Short Form (PAQ-S): a 6-item measure of alexithymia. J Affect Disord.

[68] Hwang JW, Lee GE, Woo JH, Kim SM, Kwon JY (2025). Systematic review and meta-analysis on fully automated digital cognitive behavioral therapy for insomnia. NPJ Digit Med.

[69] Erten Uyumaz B, Feijs L, Hu J (2021). A review of digital cognitive behavioral therapy for insomnia (CBT-I Apps): are they designed for engagement?. Int J Environ Res Public Health.

[70] Lee S, Park KM, Lee DH, Choi EC, Lee Y, Lee E (2025). Impact of adherence to digital cognitive behavioral therapy for insomnia effectiveness. Yonsei Med J.

[71] Lin C, Cheng C, Xia Y (2025). Internet-delivered cognitive behavioral therapy for insomnia: the future of insomnia treatment with large language models. Sleep Med X.

