# Supplementary figures and images for: SleepPathfinder: A Socratic Questioning and Self-Decision–Based Chatbot to Support User Engagement in Digital CBT-I: Usability and Feasibility Study
Source: JMIR Form Res. 2026 Jun 9;10:e79242. doi: 10.2196/79242 (PMC13249113; doi:10.2196/79242)

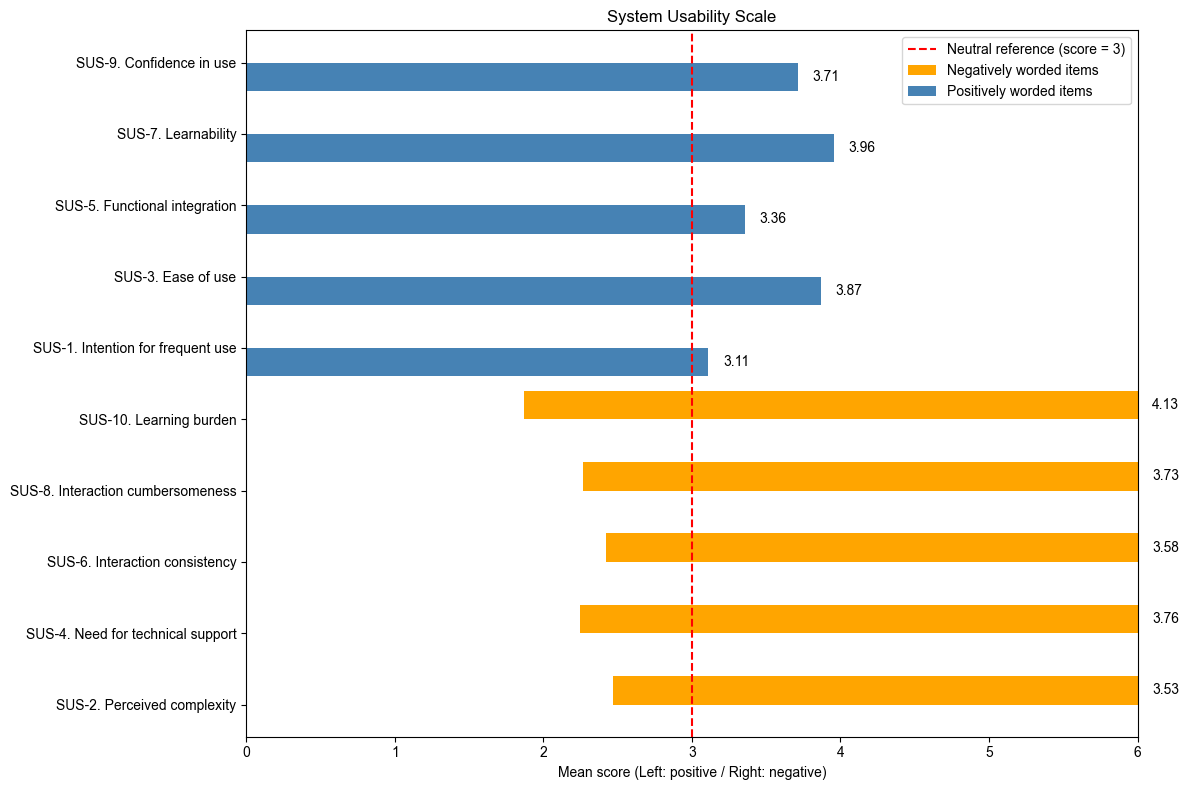

Supplement: Multimedia Appendix 2 [file formative-v10-e79242-s002.png]

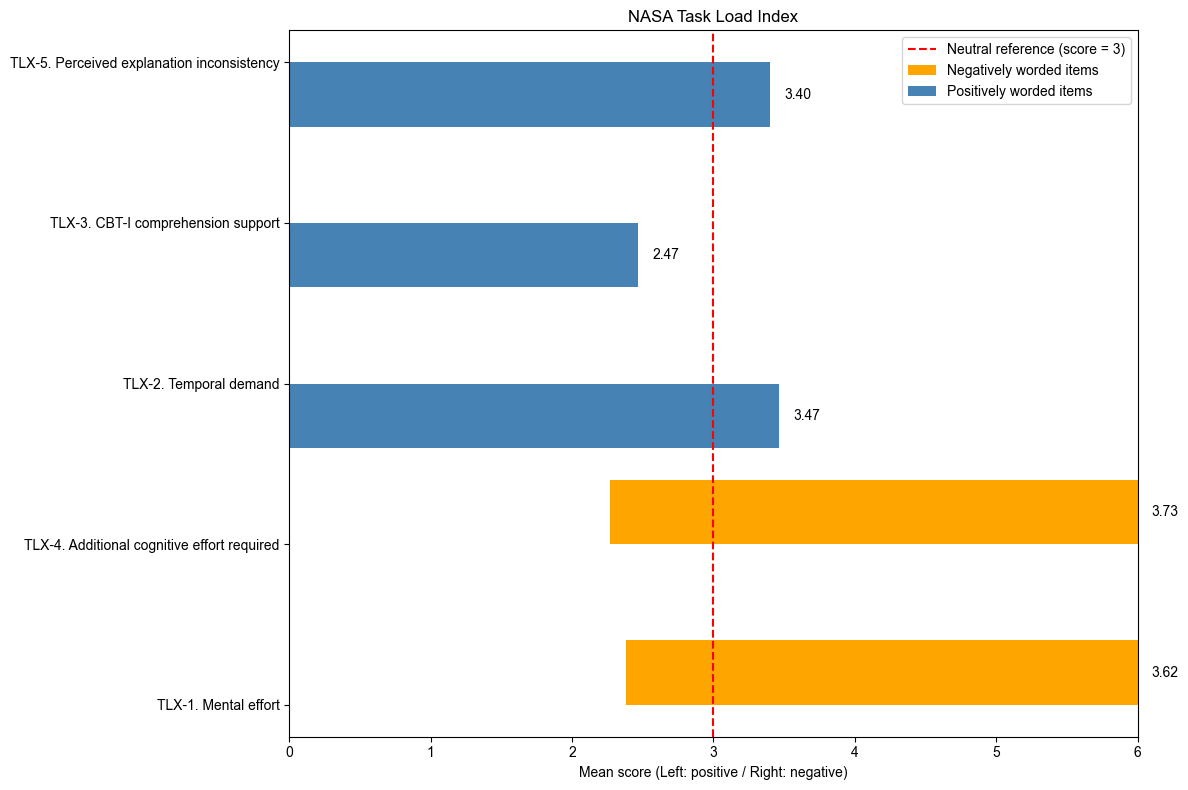

Supplement: Multimedia Appendix 3 [file formative-v10-e79242-s003.png]

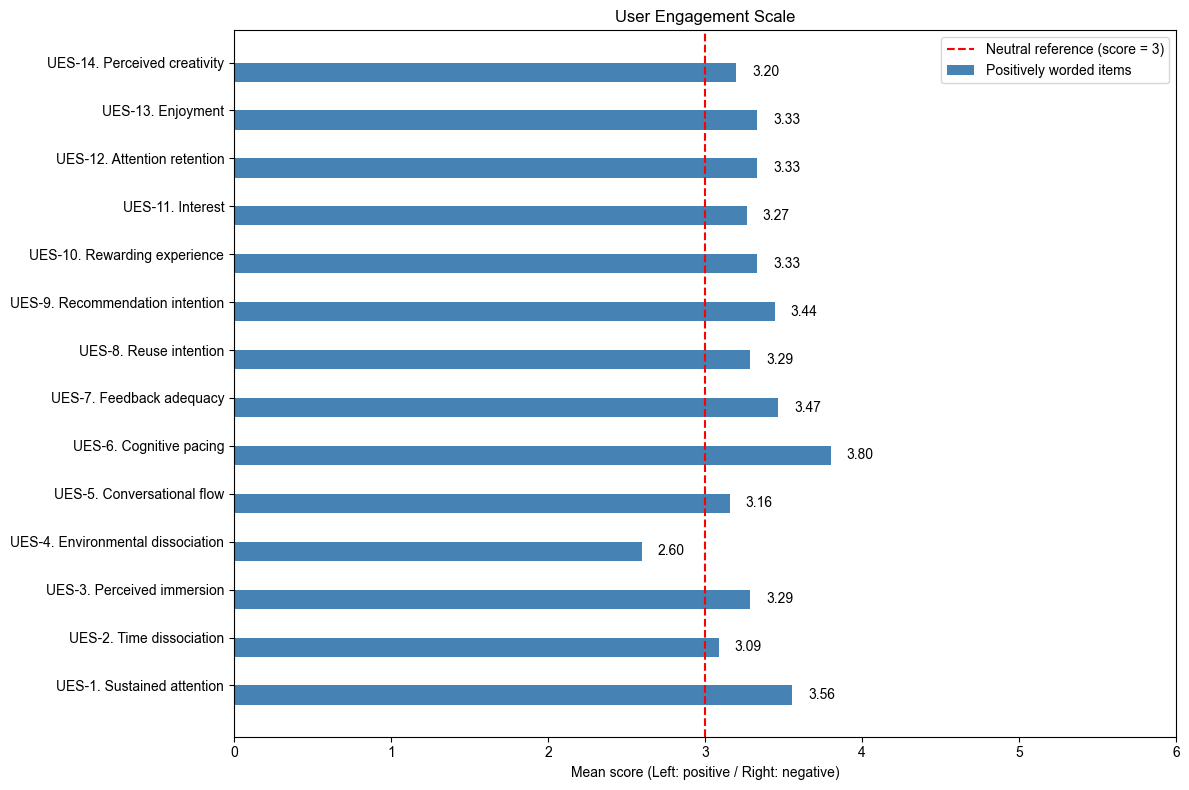

Supplement: Multimedia Appendix 4 [file formative-v10-e79242-s004.png]

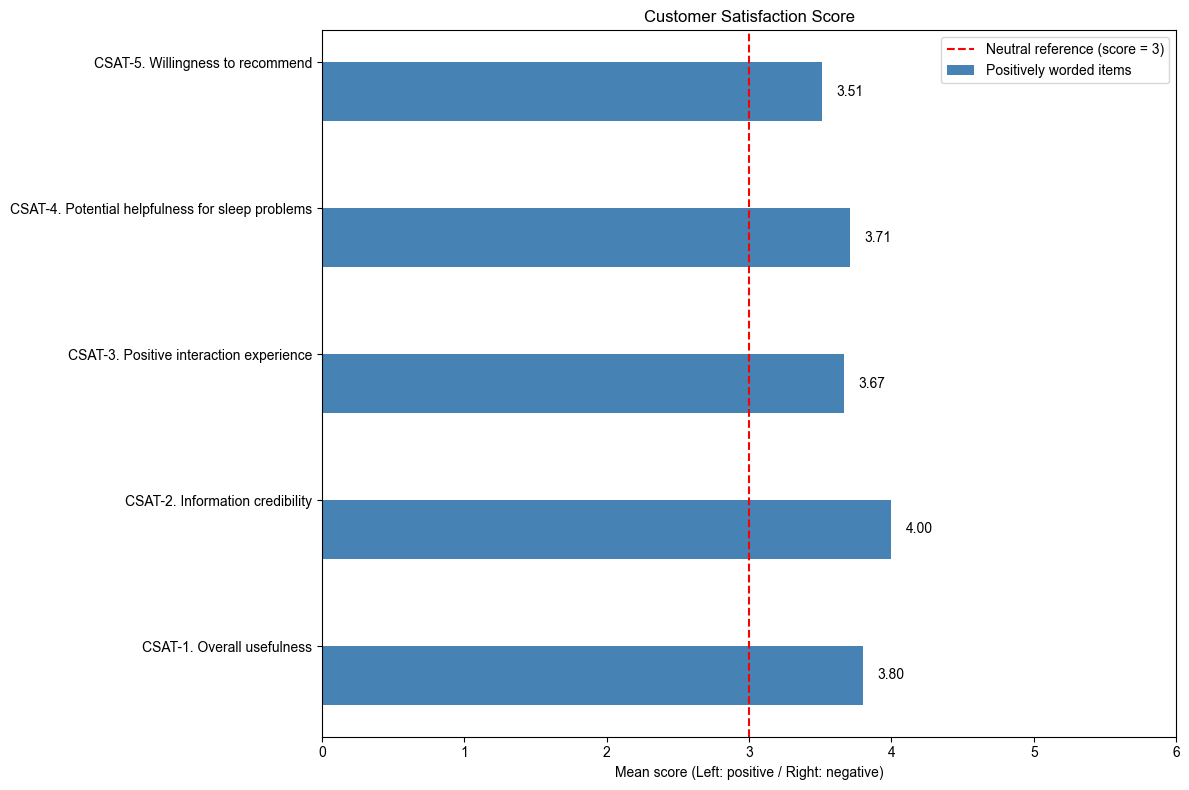

Supplement: Multimedia Appendix 5 [file formative-v10-e79242-s005.png]
