# Supplementary material for: SleepPathfinder: A Socratic Questioning and Self-Decision–Based Chatbot to Support User Engagement in Digital CBT-I: Usability and Feasibility Study
Source: JMIR Form Res. 2026 Jun 9;10:e79242. doi: 10.2196/79242 (PMC13249113; doi:10.2196/79242)
Supplement: Multimedia Appendix 6 [file formative-v10-e79242-s006.pdf]

Multimedia Appendix 6: AI-assisted exploratory thematic grouping.

Cluster 1. Satisfaction with CBT-I Information and Sleep Knowledge

- Learned about CBT-I
- Helped me reflect on my sleep pattern
- Simple interface and professional knowledge
- Provided extensive sleep-related information
- Gained new knowledge about insomnia management
- Clear explanations of sleep hygiene
- Technical terms were explained in an accessible way

Cluster 2. Positive Perception of Empathy and Human-Like Interaction

- It was good that the response began with empathy
- Empathy–question–solution felt human
- The conversation felt empathic
- Showed positive listening
- Asked specifically about my concerns
- Suggested solutions and checked satisfaction

Cluster 3. Practical Solutions and Concrete Suggestions

- Provided practical solutions
- Offered useful strategies for sleep problems
- Interpreted personal experiences based on CBT-I
- Suggested new sleep-related methods

Cluster 4. Response Speed Issues

- Too slow
- Long loading time
- Took too long to respond
- Processing time was long
- Buffering during page transition
- (Positive feedback) The response was fast
- (Positive feedback) Speed was satisfactory

Cluster 5. Repetition and Similar Responses

- Repeated similar phrases
- Duplicate responses
- Too much similar empathic wording

Cluster 6. Context Understanding and Conversational Continuity Issues

- Responses were vague
- Did not sufficiently consider previous turns
- Conversation was not smoothly connected
- Topic shifts occurred abruptly
- Slight inconsistency in responses

Cluster 7. Questioning Style and Conversational Flow

- Ended too often with questions
- Preferred answer-style responses
- Conversation felt rushed
- Wanted more clarification before solutions

Cluster 8. Robotic or Unnatural Tone

- Tone felt robotic
- Lacked natural conversational feeling
- Felt artificial
- Wanted a more friendly tone

Cluster 9. UI and Mobile Usability Issues

- Poor readability on mobile
- Screen size was too small
- Scrolling issues
- Session reset when navigating back
- Need visual emphasis (font size, color)

Cluster 10. Technical Errors and Reset Problems

- Conversation reset after several turns
- Errors interrupted the conversation
- Content was cut off
- Unexpected refresh

Cluster 11. Requests for Visual Aids and Source Transparency

- Requested visual materials
- Suggested image or video explanations
- Wanted emojis or visual elements
- Requested citation of information sources

Cluster 12. Personalization and Depth Enhancement Requests

- Referencing conversation history
- Need deeper responses based on user history
- More diverse sleep-related scenarios
- More comprehensive sleep information

- Higher-quality training data
- Regular consultation feature

Cluster 13. Structured Formatting and Information Organization

- Prefer structured format instead of long paragraphs
- Provide initial structured sleep information
- Provide answer options
- Q&A reference section

Cluster 14. Difficulty Understanding Metaphors

- Did not understand metaphorical expressions
- Excessive or inappropriate metaphors

Cluster 15. Miscellaneous

- Unexpected responses
- New topics
- General convenience comments
- None
