# Supplementary material for: SleepPathfinder: A Socratic Questioning and Self-Decision–Based Chatbot to Support User Engagement in Digital CBT-I: Usability and Feasibility Study
Source: JMIR Form Res. 2026 Jun 9;10:e79242. doi: 10.2196/79242 (PMC13249113; doi:10.2196/79242)
Supplement: Multimedia Appendix 7 [file formative-v10-e79242-s007.pdf]

**Multimedia Appendix 7.** Key focal and exploratory outcomes with effect sizes, 95% confidence intervals (CIs), and multiple-comparison adjustment. Mixed-effects models report fixed-effect estimates ($\beta$) for Group × Time interactions, whereas post-only outcomes report covariate-adjusted group effects. Benjamini–Hochberg–adjusted $q$ values are reported to control the false discovery rate ($q = 0.10$).

| **Outcome** | **Model / Contrast** | **Estimate** | **95% CI** | **Std. Effect** | $p$ **(raw)** | $q$ **(BH)** |
|---|---|---|---|---|---|---|
| **TPB Intention** (focal outcome) | Mixed-effects model, Group × Time | $\beta = -0.200$ | $[-0.683,\ 0.283]$ | $g = 0.279$ (Welch $\Delta$) | .417 | n/a |
| **HBM Perceived Severity** | Mixed-effects model, Group × Time | $\beta = 0.467$ | $[0.078,\ 0.855]$ | $g = -0.808$ (Welch $\Delta$) | .019 | .3703 |
| HBM Perceived Susceptibility | Mixed-effects model, Group × Time | $\beta = -0.044$ | $[-0.586,\ 0.497]$ | $g = 0.055$ | .872 | .9461 |
| HBM Self-Awareness | Mixed-effects model, Group × Time | $\beta = 0.244$ | $[-0.305,\ 0.794]$ | $g = -0.299$ | .383 | .7890 |
| TPB Outcome Beliefs | Mixed-effects model, Group × Time | $\beta = -0.133$ | $[-0.705,\ 0.438]$ | $g = 0.157$ | .647 | .8525 |
| TPB Subjective Norms | Mixed-effects model, Group × Time | $\beta = 0.433$ | $[-0.250,\ 1.116]$ | $g = -0.427$ | .214 | .7354 |
| TPB Self-Efficacy | Mixed-effects model, Group × Time | $\beta = -0.022$ | $[-0.639,\ 0.595]$ | $g = 0.024$ | .944 | .9461 |
| **SDT Autonomy (post)** | ANCOVA, covariate-adjusted post | – | – | $\eta^2 = 0.168$ | .073 | .7354 |
| UEQ-S Hedonic Quality (post) | ANCOVA, covariate-adjusted post | – | – | $\eta^2 = 0.096$; $g = 0.435$ | .183 | .7354 |
| UEQ-S Total (post) | ANCOVA / $t$ test | – | – | $g = 0.165$ | .376 | .8525 |
| **Stage of Change** | Ordered logistic regression, post | OR = 3.16 | $[1.12,\ 8.92]$ | – | .0296 | n/a |
